# Effect of Engagement With Digital Interventions on Mental Health Outcomes: A Systematic Review and Meta-Analysis

**DOI:** 10.3389/fdgth.2021.764079

**Published:** 2021-11-04

**Authors:** Daniel Z. Q. Gan, Lauren McGillivray, Jin Han, Helen Christensen, Michelle Torok

**Affiliations:** Black Dog Institute, University of New South Wales, Sydney, NSW, Australia

**Keywords:** digital mental health, eHealth, mHealth, systematic review, meta-analysis

## Abstract

Digital mental health interventions (DMHIs) present a promising way to address gaps in mental health service provision. However, the relationship between user engagement and outcomes in the context of these interventions has not been established. This study addressed the current state of evidence on the relationship between engagement with DMHIs and mental health outcomes. MEDLINE, PsycINFO, and EmBASE databases were searched from inception to August 1, 2021. Original or secondary analyses of randomized controlled trials (RCTs) were included if they examined the relationship between DMHI engagement and post-intervention outcome(s). Thirty-five studies were eligible for inclusion in the narrative review and 25 studies had sufficient data for meta-analysis. Random-effects meta-analyses indicated that greater engagement was significantly associated with post-intervention mental health improvements, regardless of whether this relationship was explored using correlational [*r* = 0.24, 95% CI (0.17, 0.32), *Z* = 6.29, *p* < 0.001] or between-groups designs [Hedges' *g* = 0.40, 95% CI (0.097, 0.705), *p* = 0.010]. This association was also consistent regardless of intervention type (unguided/guided), diagnostic status, or mental health condition targeted. This is the first review providing empirical evidence that engagement with DMHIs is associated with therapeutic gains. Implications and future directions are discussed.

**Systematic Review Registration:** PROSPERO, identifier: CRD 42020184706.

## Introduction

Mental illness is a worldwide public health concern with an overall lifetime prevalence rate of ~14%, and accounting for 7% of the overall global burden of disease (1). The estimated impact of mental illness on quality of life has progressively worsened since the 1990's, with the number of disability-adjusted life years attributed to mental illness estimated to have risen by 37% between 1990 and 2013 (2). This rising mental health burden has prompted calls for the development of alternative models of care to meet the increasing treatment needs, which is unlikely to be able to be adequately serviced through face-to-face services into the future (3).

Digital mental health interventions (DMHIs) are one alternative model of care with enormous potential as a scalable solution for narrowing this service provision gap. By leveraging technology platforms (e.g., computers and smartphones) well-established psychological treatments can be delivered directly into people's hand with high fidelity and no-to-low human resources, empowering individuals to self-manage mental health issues (4). The anonymity, timeliness of access, and flexibility afforded by DMHIs also circumvents many of the commonly identified structural and attitudinal barriers to accessing care such as cost, time, or stigma (5). While there is a growing body of evidence supporting that DMHIs can have significant small-to-moderate effects on alleviating or preventing symptoms of mental health disorders (6–10), low levels of user engagement have been reported as barriers to both optimal efficacy and adoption into health settings and other translational pathways. Health professionals will require further convincing that digital interventions are a viable adjunct or alternative to traditional therapies before they are willing to advocate for patient usage as a therapeutic adjunct (11). Low engagement—defined as suboptimal levels of user access and/or adherence to an intervention (12)—is touted as one of the main reasons why the potential benefits of these interventions remain unrealized in the real world (13, 14).

Recognizing the need to promote better engagement with digital interventions, several review studies have sought to establish both the nature of engagement with DMHIs, and the effectiveness of various engagement strategies to improve uptake of, and adherence to, DMHIs. A review of empirical studies has found collectively low rates of DMHI completion, with over 70% of users failing to complete all treatment modules, and more than 50% disengaging before completing half of all treatment modules (15). Reviews of the effectiveness of various engagement strategies have shown that such strategies can have a positive impact on engagement with, and efficacy of, digital interventions. Reminders, coaching, and tailored feedback delivered *via* telephone or email have been found to have modest to moderate effects toward increasing engagement with digital interventions targeting physical and mental health outcomes, compared to if no strategy was used (16). In addition, a review of efficacy studies of smartphone apps for depression and anxiety found that apps which incorporated more elements aimed at promoting user engagement had larger effect sizes (17).

Though these prior reviews are undoubtedly important to accelerating our understanding of how the benefits of DMHIs might be realized in real world settings, it may be premature to invest substantial effort in engagement strategies. To date, only one prior systematic review (18) has explored the effect of engagement on the effectiveness of digital interventions. This review was limited to a narrative synthesis, citing heterogeneity in how engagement is operationalized (e.g., number of logins, module completion, frequency of use, and time spent in the intervention) as a barrier to meta-analysis. Accordingly, no meta-analyses have yet empirically examined the pooled strength of the association between DMHI engagement and its impacts on mental health outcomes. Despite the lack of meta-analytic evidence, it has been widely assumed that the association between face-to-face treatment engagement and treatment success extends to these tools by virtue of the extensive replication of this relationship in the general intervention literature (19). However, extrapolating research findings from face-to-face therapies to their digital equivalents may not be appropriately comparable because differences in how content is delivered (e.g., *via* developing human relationships) is likely to affect both engagement and associated treatment responses (20).

The increasing number of individual studies examining the engagement—mental health outcome association published in recent years, now enables a review and quantification of the literature. To address this gap in knowledge, and justify the need for the development of engagement strategies, the primary purpose of this meta-analysis is to examine the relationship between level of engagement and change in mental health outcomes in the context of digital mental health interventions. Our primary hypothesis is that poor engagement is associated with non-significant changes in mental health outcomes, given that individual studies suggest that users who engage poorly with DMHIs derive limited treatment benefit (21, 22). This is the first meta-analysis to quantify the association between engagement and primary mental health outcome measures in respect to digital interventions, and as such, extends previous work which has been constrained by a small pool characterized by substantial heterogeneity in how engagement with DMHIs is measured.

## Methods

### Search Strategy

This study is registered with PROSPERO (CRD 42020184706) and adheres to the Preferred Reporting Items for Systematic Reviews and Meta-analyses (PRISMA) reporting guidelines (23).

A systematic search was undertaken in three online academic databases from inception to articles indexed as of August 1, 2021: MEDLINE (from 1946), PsycINFO (from 1806), and EmBASE (from 1947). A test set of five papers meeting inclusion criteria (described later) was obtained *via* manual search. From these papers, a set of primary search terms were developed using Medical Subject Heading (MeSH) terms and centered around four search blocks: (i) engagement/ adherence, (ii) digital interventions, (iii) mental health, and (iv) study design. This search strategy was tested in MEDLINE, where it achieved 100% sensitivity against the initial test set, and was subsequently adapted for use in the other databases. A manual ancestry search of reference lists of eligible studies identified from the database search was also conducted to identify other relevant studies that could have been missed. The search terms can be found in the Supplementary Material (p. 1).

Following removal of duplicate records, titles and abstracts were independently coded for relevance by two authors (DZQG and MT). Screening of full-text records was performed in a similar way (DZQG and LM). Disagreements were resolved *via* mutual discussion. Inter-rater consensus was acceptable for both title/abstract (κ = 0.86, *p* < 0.001) and full-text screening (κ = 0.80, *p* < 0.001).

### Selection Criteria

Studies were eligible for inclusion if they were original or secondary analyses of randomized controlled trial (RCT) evaluations of digital interventions that were specifically designed for a mental health issue and which quantitatively examined the relationship between engagement and mental health. Only digital interventions that delivered manualised therapeutic content to users *via* a digital platform (e.g., smartphone, tablet, and computer) were included. The interventions could be unguided (self-directed, used independently without support or guidance by a trained health professional) or guided (health professional-assisted) and differences in these approaches were accounted for by analyzing them separately in the sub-analyses. The rationale for including only RCT studies was to understand if the intervention being tested had a main effect on efficacy to then be able to contextualize the impact of engagement. Non-experimental studies are unable to causally establish efficacy, making it redundant as to whether user engagement improves outcomes for an intervention that we are unable to determine works or not. Engagement was defined as any objective indicator used to quantify the extent of intervention use. Continuous measures of engagement were generally focused on the extent of content accessed (e.g., number of modules, sessions, or lessons completed), or the extent of intervention-related activity (e.g., number of logins or visits, time spent, specific activities or exercises completed, number of online interactions with therapists or peers). Categorical measures of engagement focused on the percentage of users who downloaded, logged-in, and/or completed the intervention.

Studies were excluded if they were not peer-reviewed journal articles (e.g., dissertations, conference presentations, etc.), in a language other than English, or if they did not investigate the relationship between engagement and mental health outcomes. No restrictions were placed on the target population, setting, type of mental health condition targeted, intervention type, or language that the interventions were delivered in.

To improve comparability and reduce heterogeneity, studies were excluded if the digital intervention was (i) targeted at caregivers or health professionals (i.e., gatekeepers) rather than individuals with the mental health condition of interest, (ii) comprised solely of activities (e.g., journal and mood tracking) or psychoeducational material without any therapeutic content, or (iii) that were delivered by other digital means (e.g., fully text-based interventions, pre-recorded videos, and DVD).

### Data Extraction and Analysis

Key study information was extracted and recorded in a custom spreadsheet by three authors (DZQG, MT, and LM). One author (DZQG) extracted data for all the included papers. Accuracy of the extraction was checked by another author (LM). Any differences were resolved through discussion, and in cases where no consensus was reached, a third author (MT) was consulted. Such information included the (i) study design, (ii) intervention characteristics, (iii) target population and recruitment, (v) how engagement was measured, (vi) primary mental health condition targeted, and (vii) findings pertaining to the relationship between DMHI engagement and the primary mental health outcome. Corresponding authors of studies were contacted by email if more information was needed to determine eligibility. Based on previous research (18), studies were expected to vary in the engagement measures and mental health outcomes reported. A narrative review was therefore undertaken to ensure a systematic discussion of the findings from all studies. Studies which utilized similar measures of engagement and which employed similar analytical techniques to test the association between engagement and outcome(s) were pooled together for meta-analysis.

Random effects meta-analyses with the Pearson correlation coefficient (*r*) were used to examine the relationship between engagement strategies and mental health outcomes. This was different from the effect size measure (Cohen's *d*) stated in the protocol. However, *r* was determined to be more appropriate as it was commonly reported by the included studies. Corresponding authors for 33 of the 35 studies were contacted for statistical or other study data. If correlation coefficients could not be obtained, estimates of *r* were derived based on the data available. If non-parametric correlations were reported, estimates were computed using formulae provided by Gilpin (24); if beta regression coefficients were reported, estimates were computed using formulae proposed by Peterson and Brown (25). All correlations were standardized such that a positive coefficient indicated that greater DMHI engagement was associated with improvements in mental health (i.e., reduced symptom severity) at post-intervention.

Based on the available data, two separate analyses were conducted to address our a priori hypothesis that better engagement with digital interventions is associated with greater improvement in mental health outcomes at post-intervention. Two analyses were needed because effect sizes of studies using between-group vs. correlational designs cannot be combined (26). One meta-analysis was performed for studies which examined this association using a correlational design, while the other was conducted for studies which investigated this relationship using a between-groups design. Quantitative data for each meta-analysis were summarized using the Pearson correlation coefficient *r* or the Hedges' *g* statistics (27), respectively. For studies where data were summarized using correlation coefficients, all analyses were performed on the transformed Fisher's *z*-values, and subsequently transformed back to *r* to yield an overall summary correlation (27). Subgroup analyses were planned a priori and included comparisons between (i) level of assistance with engagement (guided or unguided interventions), (ii) user mental health severity (diagnosed or non-diagnosed), and (iii) primary mental health target (depressive- or anxiety-related symptoms).

Study heterogeneity was evaluated for each meta-analysis using the *I*^2^ statistic, with thresholds of 25, 50, and 75% denoting low, moderate, and high levels of heterogeneity, respectively (28). Ninety-five percent confidence intervals (CIs) for *I*^2^ values were computed using formulae proposed by Borenstein et al. (29). For meta-analyses with a sufficient number of included studies (i.e., ≥10 studies), publication bias was assessed using funnel plots and Egger's regression test (30). All analyses were performed using Comprehensive Meta-Analysis version 3.0 (31).

Risk of bias was assessed using Version 2 of the Cochrane risk-of-bias tool for randomized trials (RoB 2) (32). The RoB 2 comprises five domains and an overall risk domain, each of which were scored against a three-point rating scale corresponding to “low,” “some,” or “high” risk of bias. Ratings were independently conducted by two pairs of coders (40% by DZQG and LM, and 60% by DZQG and JH). Discrepancies were resolved through mutual discussion. An overall agreement rate of 93.3% was reached.

## Results

**Figure 1:**
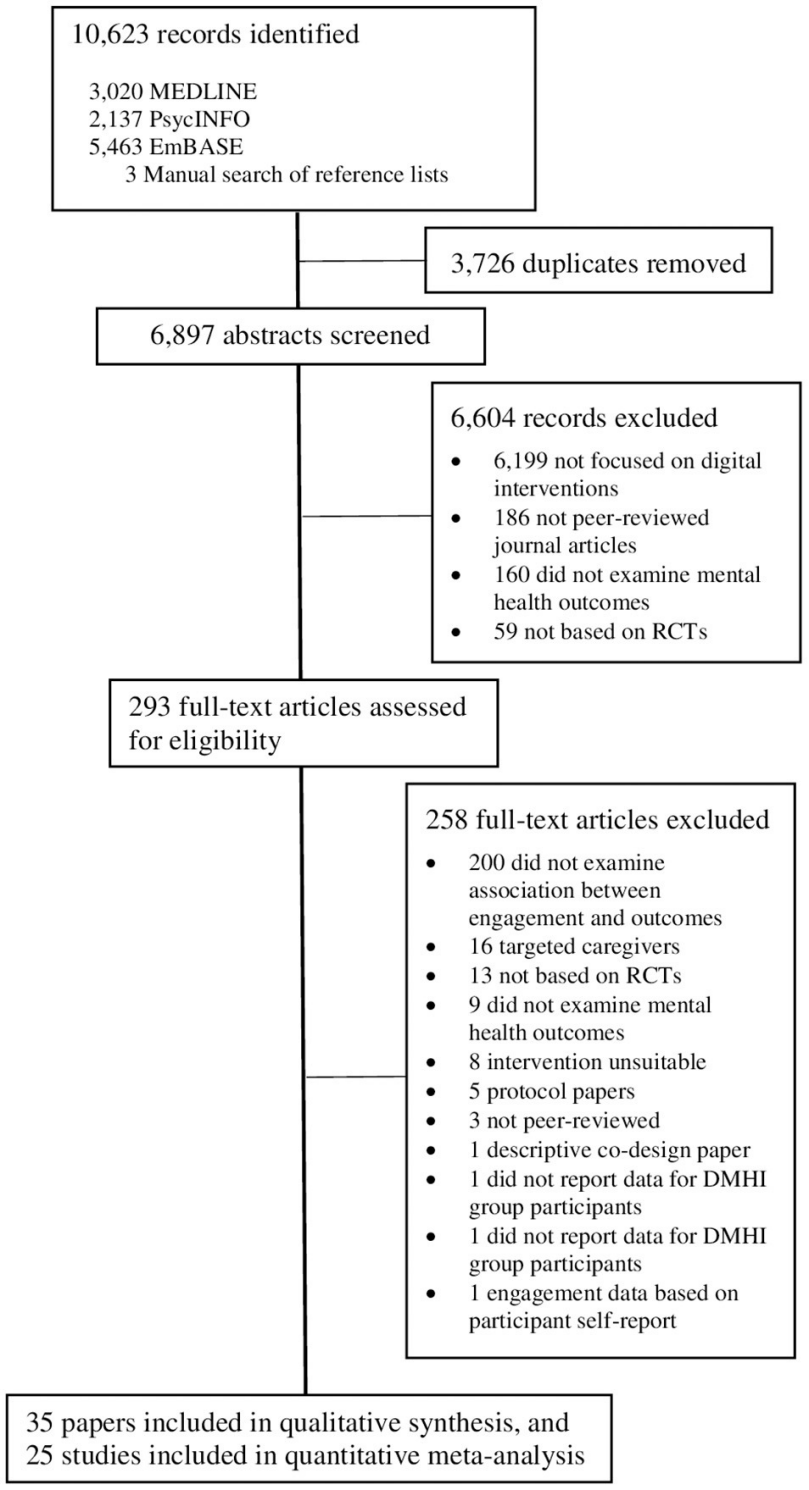
PRISMA diagram for study selection process.

The search yielded a total of 10,623 articles. Following removal of duplicates and non-eligible studies, 35 unique studies were identified that met inclusion criteria (Figure 1). All studies were included in the narrative synthesis, and 25 of the 35 were included in the meta-analysis. Ten studies could not be included in the meta-analysis because study authors were either unable to provide the data required for effect size calculation (*n* = 7) or were uncontactable (*n* = 3).

### Study Characteristics

Table 1 summarizes key information from the 35 studies, which provided baseline data on a total of 4,484 participants who were assigned to receive the digital intervention, and 8,110 participants in total (intervention and control conditions). Consistent with the aims of the present study, from herein we only report data for participants assigned to the intervention condition. Intervention condition sample sizes ranged from 22 to 542 participants (*Mdn* = 81). Mean participant ages ranged from 11.0 (*SD* 2.57) to 58.4 (*SD* 9.0) years. Proportion of female participants ranged from 5.3 to 100% (*Mdn* = 68.8%). Most studies were conducted with adults (aged 18 years and above; 30 studies; 85.7%). Duration between baseline and post-intervention assessments for mental health outcomes ranged from 3 to 14 weeks (*Mdn* = 9). The studies were carried out in middle to high income countries across four continents: Europe (*n* = 21; 60%), North America (*n* = 9; 25.7%), Australia (*n* = 3; 8.6%), and Asia (*n* = 2, 5.7%). Most of the interventions were either fully or partially based on cognitive-behavioral therapy (27 studies, 80%). Digital interventions were designed to address a range of mental health symptoms; the most common symptoms targeted were anxiety (10 studies, 28.6%), depression, (nine studies, 25.7%), and psychological distress/recovery (nine studies, 25.7%). Thirty (85.7%) of the DMHIs were online programs accessed primarily *via* computer, while the remaining five were app-based interventions accessed *via* smartphones. The number of modules/activities/sessions completed was the most commonly reported engagement measure (33 studies, 94.3%). Other engagement metrics included number of logins (five studies; 14.3%), time spent using the intervention (five studies, 14.3%), and other actions performed in response to DMHI content, such as emails to therapist, interactions with other users (four studies, 11.4%). Ten studies (28.6%) reported on more than one measure of engagement.

**Table 1:** Characteristics of the included studies.

**Author(s), year, country (References)**	**Study type**	**Theoretical model**	**Duration of intervention**	**Sample mental health characteristics**	**Target population**	**Baseline sample size**	**Primary mental health outcome**	**Engagement metric(s)**
**Unguided interventions (17 studies)**								
Andersson et al., 2005, Sweden (33)	RCT	Internet-administered self-help (CBT)	5 modules / 8 weeks	Mild to moderate symptoms of depression; based on self-report.	Adults M_age_ = 36.4 (11.5) 78% female	57	Depression	Modules completed (+)
Berger et al., 2011, Switzerland (34)	RCT	Internet-based self-help (CBT)	5 lessons / 10 weeks	Fulfill diagnostic criteria for social anxiety disorder.	Adults M_age_ = 37.2 (11.2) 53.1% female	81	Anxiety (social phobia)	Lessons completed (+) Time spent (+) No. of clicks (+) No. of diary entries (+)
Berger et al., 2017, Switzerland (35)	RCT	Velibra (CBT)	6 modules / 9 weeks	Fulfill diagnostic criteria for anxiety-related disorders.	Adults M_age_ = 42.0 (12.1) 70.5% female	70	Psychological distress	Modules completed (ns) Time spent (ns)
Bolier et al., 2013, Netherlands (36)	RCT	Psyfit (Positive psychology)	6 modules / 8 weeks	Mild to moderate symptoms of depression; based on self-report.	Adults M_age_ = 43.5 (11.7) 79.7% female	143	Psychological distress	Lessons completed (+)
Bruehlman-Senecal et al., 2020, USA (37)	RCT	Nod (Positive psychology, Mindfulness-based self-compassion, CBT)	No module information / 4 weeks	No mental health-related inclusion criteria.	1st year college students. M_age_ = 18.7 (0.33) 51% female	100	Loneliness	App pages visited (ns) Social challenges completed (+) Reflections accessed (ns)
Calear et al., 2013; Australia (38)	Secondary analysis	MoodGym (CBT)	5 modules / 9 weeks	No mental health-related inclusion criteria.	School children M_age_ = 14.56 (0.68) 63.1% female	530	Anxiety (general) Depression	Exercises completed (ns for both outcomes)
Casey et al., 2017; Australia (39)	RCT	Improving the Odds (CBT)	6 modules / 6 weeks	Fulfill diagnostic criteria for gambling disorder.	Adults M_age_ = 14.56 (0.68) 79.7% female	60	Gambling cognitions	Sessions completed (+)
Forand et al., 2017, Netherlands (40)	Secondary analysis	Color Your Life (CBT)	9 sessions / 9 weeks	Mild to moderate symptoms of depression; based on self-report.	Adults M_age_ = 44.7 (11.3)	200	Depression	Modules completed (+)
Heckendorf et al., 2019, Germany (41)	RCT	GET.ON Gratitude (Gratitude)	5 modules / 5 weeks	Elevated levels of repetitive negative thinking; based on self-report.	Adults M_age_ = 42.3 (10.6) 54.5% female	132	Repetitive negative thinking	Activities completed (+)
Hensel et al., 2019, Canada (42)	RCT	Big White Wall (BWW)	No module information / 13 weeks (3 months)	Attending outpatient mental health programs. No diagnostic information.	Adults M_age_ = 41.5 (13.4) 73% female	542	Psychological recovery	No. of logins (ns)
Krieger et al., 2019, Switzerland (43)	RCT	Mindfulness-Based Compassionate Living (MBCL)	7 modules / 8 weeks	Elevated levels of self-criticism; based on self-report.	Adults M_age_ = 38.0 (12.0) 84.7% female	59	Self-compassion	Modules completed (+) Time spent in program (ns)
Levin et al., 2016, USA (44)	RCT	Acceptance and Commitment Training on College Life (ACT-CL)	2 modules / 3 weeks	No mental health information.	Young adults (college students) M_age_ = 21.6 (5.48) 76.9% female	110	Psychological distress	Sessions completed (ns) No. of logins (ns) Response to exercises (ns)
Luo et al., 2021, China (45)	RCT	eBody Project	6 modules / 6 weeks	Body dissatisfaction; based on self-report	Youths and young adults M = 17.36 (1.37) 100% female	191	Body dissatisfaction	Sessions completed (ns)
Twomey et al., 2014, Ireland (46)	RCT	MoodGym (CBT)	6 modules / 4 weeks	Self-reported symptoms of stress, anxiety, or depression	Adults M_age_ = 37.3 (10.9) 89.3% female	80	Psychological distress	Sessions completed (ns)
Wilson et al., 2018, USA (47)	RCT	Goalistics Chronic Pain Management Program (CBT)	2 h per week / 8 weeks (No. modules not stated)	Patients with chronic non-cancer pain and receiving treatment for opioid use	Adults M_age_ = 45.5 (12.4) 48.4% female	31	Pain self-efficacy	Activities completed (+)
Wilson et al., 2018, USA (48)	RCT	Think Clearly About Depression (CBT)	14 modules / 8 weeks	Self-reported symptoms of depression of moderate severity	Adults M_age_ = 45.7 (12.8) 91% female	22	Depression	Activities completed (ns)
Zeng et al., 2020, China (49)	Secondary analysis	Run4Love (CBT)	65 activities / 12 weeks	Individuals with HIV with self-reported elevated symptoms of depression	Adults M_age_ = 28.0 (5.8) 5.3% female	150	Depression	Item completion rate (+) Frequency of items completed (+) Time spent (ns)
**Guided interventions (14 studies)**								
Ben-Zeev et al., 2018, USA (50)	RCT	FOCUS (CBT)	12 weeks	Fulfill diagnostic criteria for schizophrenia-related, bipolar, or depressive disorder.	Adults M_age_ = 49.0 (9.8) 42% female	82	Psychological recovery	Weeks completed (+)
Berger et al., 2014; Switzerland (51)	RCT	Internet-Based Self-Help (CBT)	8 sessions / 8 weeks	Fulfill diagnostic criteria for anxiety-related disorders.	Adults M_age_ = 34.7 (11.3) 56.8% female	88	Anxiety (general)	Sessions completed (+)
Cillessen et al., 2020, Netherlands (52)	Secondary analysis	Mindfulness-based cognitive therapy (MCBT)	9 sessions / 9 weeks	Cancer patients with mild psychological distress; based on self-report.	Adults M_age_ = 52.0 (10.2)	125	Psychological Distress	Exercises completed (+) Emails sent (ns) Mean time logged in (ns) Total time logged in (ns) No. of logins (ns)
El Alaoui et al., 2013, Sweden (53)	Secondary analysis	ICBT	10 modules / 10 weeks	Fulfill diagnostic criteria for panic disorder.	Adults M_age_ = 33.8 (9.7)	50	Anxiety (panic disorder)	Modules completed (ns)
Hedman et al., 2015, Sweden (54)	Secondary analysis	ICBT (CBT)	12 modules / 12 weeks	Fulfill diagnostic criteria for hypochondriasis.	Adults M_age_ = 41.7 (13.6) 85% female	79	Anxiety: health-related	Modules completed (+)
Hedman et al., 2013, Sweden (55)	Secondary analysis	ICBT (CBT)	12 modules / 12 weeks	Fulfill diagnostic criteria for hypochondriasis.	Adults M_age_ = 39 (9.7) 74% female	81	Anxiety: health-related	Modules completed (+)
Lenhard et al., 2017, Sweden (56)	RCT	BiP for OCD (CBT)	12 chapters / 12 weeks	Fulfill diagnostic criteria for OCD.	Youth M_age_ = 15.0 (1.66) 41% female	33	OCD severity	Chapters completed (ns)
Lundgren et al., 2016, Sweden (57)	RCT	ICBT (CBT)	7 modules / 9 weeks	Mild to moderate symptoms of depression; based on self-report	Adults M_age_ = 63.6 (13.9) 60% female	25	Depression	Modules completed (ns) No. of logins (+)
Moberg et al., 2019, USA (58)	RCT	Pacifica (CBT, Mindfulness)	35 lesson-activity pairs / 5 weeks	Self-reported, mild to moderate symptoms of depression or anxiety.	Adults M_age_ = 30.2 (10.9) 75% female	253	Depression Anxiety	No. logins (ns for both) Activities completed (ns for both)
Norlund et al., 2018, Sweden (59)	RCT	ICBT	10 modules / 14 weeks	Patients with myocardial infarction with mild symptoms of depression or anxiety.	Adults M_age_ = 58.4 (9.0) 37.6% female	117	Psychological distress	Homework assignments completed (ns)
Schlosser et al., 2018, USA (60)	RCT	PRIME (personalized real-time intervention for motivational enhancement; CBT)	NA / 12 weeks (at least 1 login per week)	Meet criteria for schizophrenia- related disorders	Adults M_age_ = 24.3 (2.6) 40% female	22	Motivation in schizophrenia	No. coach interactions (ns) No. peer interactions (ns) Goals completed (ns)
Spence et al., 2017, Australia (61)	RCT	BRAVE-Online (CBT)	10 sessions / 10 weeks	Meet criteria for social anxiety disorder.	Children and youth M_age_ = 11.0 (2.57) 62.1% female	95	SAD diagnostic severity	Sessions completed (ns)
Stjerneklar et al., 2019, Denmark (20)	Secondary analysis	ChilledOut Online (CBT)	8 modules / 14 weeks	Meet criteria for an anxiety disorder.	Youth M_age_ = 15.2 (1.33) 78% female	64	Anxiety	Modules completed (ns)
Todd et al., 2014, UK (62)	RCT	Living With Bipolar (LWB; CBT)	15 modules / 12 weeks	Self-reported symptoms of bipolar disorder.	Adults M_age_ = 42.0 (10.4) 74% female	59	Quality of life (bipolar disorder)	Modules completed (ns)
**Guided and unguided interventions (4 studies)**								
Fuhr et al., 2018, Germany (63)	Secondary analysis	Deprexis (CBT)	10 modules / 12 weeks	Mild to moderate symptoms of depression; based on self-report.	Adults M_age_ = 42.8 (11.0) 68.8% female	485	Depression	Sessions completed (+) Time spent (+)
Levin et al., 2021, USA (64)	RCT	Online Acceptance Commitment Therapy (ACT)	12 sessions / 6 weeks	Psychological distress; based on self-report.	Young adults (college students) with self-reported M_age_ = 22.3 (5.08) 72.4% female	136	Psychological distress	Sessions completed (ns)
Mira et al., 2017, Spain (65)	RCT	Sonreír es Divertido (Cognitive therapy, positive psychology)	8 modules / 12 weeks	Mild to moderate symptoms of depression; based on self-report	Adults M_age_ = 35.2 (9.7) 65% female	80	Depression	Modules completed (+)
Oromendia et al., 2016, Spain (66)	RCT	Free from Anxiety (CBT)	8 modules / 8 weeks	Meet criteria for panic disorder.	Adults M_age_ = 39.4 (8.5) 73.1% female	52	Anxiety (panic disorder)	Modules completed (+)

*+Greater engagement associated with post-intervention mental health improvements*.

*ns, no association between engagement measure and post-intervention symptoms*.

### Risk of Bias

Assessment of the methodological quality of studies on the Cochrane Risk of Bias 2.0 tool found most studies to have some level of potential bias (Supplementary Material, p. 2). Selective reporting was identified to be the largest source of bias, with 28 studies (80.0%) not reporting sufficient information—such as a prospectively published trial protocol—to rule out bias in this domain. Risk of outcome measurement bias was the second most common source of potential bias, with 19 studies (54.3%) reporting that outcome assessors—usually study participants themselves—were aware of the intervention received by study participants. Participants in 16 studies (45.7%) were not blinded to their assigned intervention. Most studies had sound random sequence generation and allocation concealment processes, and employed analytical techniques to minimize bias in missing post-intervention outcome data.

### Narrative Synthesis of the Effect of Engagement on Mental Health Outcomes

Of the 35 studies, 14 (40%) reported evidence that greater engagement with DMHIs was associated with statistically significant improvements in mental health symptoms at post-intervention, and this was consistent across all engagement measures used if multiple measures were used in a single study (33, 34, 36, 39, 40, 50–52, 54, 55, 61, 63, 65, 66). Five studies (17.1%) reported mixed findings, where improvements in the primary mental health outcome were associated with some, but not all, measures of engagement (37, 43, 47, 49, 57). Sixteen studies (45.7%) did not find any significant association between engagement and post-intervention mental health outcomes (20, 35, 38, 41, 42, 44–46, 48, 53, 56, 58–60, 62, 64).

#### Unguided vs. Guided Interventions

Seventeen studies evaluated digital interventions that were unguided (33–49). Of these, nine reported a positive association between engagement and mental health outcomes (33, 34, 36, 39–41, 43, 47, 49) while eight did not find a significant association (35, 37, 38, 42, 44–46, 48). Fourteen studies examined DMHIs that were guided (20, 50–62). Among these studies, six reported a positive association between engagement and mental health outcomes (50–52, 54, 55, 57) while eight did not find a significant association (20, 53, 56, 58–62). The remaining four studies included participants who used both unguided and guided versions of the same digital intervention (63–66). Of these studies, three reported a positive association between engagement and mental health outcomes (63, 65, 66) while one did not find a significant association (64).

#### Mental Health Diagnostic Status

In 12 of the studies, participants' mental health status were confirmed with formal diagnostic instruments. These ranged from anxiety-related disorders (20, 34, 35, 51, 53–55, 61, 66), schizophrenia-related disorders (60), gambling disorder (39), and obsessive-compulsive disorder (56). Of these studies, six reported a positive association between engagement and the primary mental health outcome (34, 39, 51, 54, 55, 66) while the other six did not find a significant association (20, 35, 53, 56, 60, 61). In the remaining 23 studies, participants were not formally diagnosed with a mental health condition but were screened for symptoms indicative of mental disorders. Of these studies, 12 reported a positive association between engagement and the primary mental health outcome (33, 36, 40, 41, 43, 47, 49, 50, 52, 57, 63, 65) while the other 11 did not find a significant association (37, 38, 42, 44–46, 48, 58, 59, 62, 64).

#### Specific Mental Health Outcomes

##### Anxiety

Ten studies (20, 34, 38, 51, 53–55, 58, 61, 66) investigated the relationship between engagement and anxiety-related symptoms. Of these, five reported a positive association between engagement and post-intervention symptoms (34, 51, 54, 55, 66) while five did not find any significant association (20, 38, 53, 58, 61).

##### Depression

Nine studies (33, 38, 40, 48, 49, 57, 58, 63, 65) investigated the relationship between engagement and depression-related symptoms. Of these, six reported a positive association between engagement and post-intervention symptoms (33, 40, 49, 57, 63, 65) while three did not find any significant association (38, 48, 58).

##### Psychological Distress

Nine studies (35, 36, 42, 44, 46, 50, 52, 59, 64) investigated the relationship between engagement and psychological distress or psychological recovery. Of these, three reported a positive association between engagement and post-intervention symptoms (36, 50, 52) while six did not find any significant association (35, 42, 44, 46, 59, 64).

##### Other Mental Health Outcomes

Finally, nine studies reported associations between engagement and other primary mental health outcomes. These outcomes include loneliness (37), gambling symptoms (39), repetitive negative thinking (41), self-compassion (43), OCD symptoms (56), body dissatisfaction (45), motivation in schizophrenia (60), quality of life in bipolar disorder (62), and pain self-efficacy (47). Of these, five studies reported a positive association between engagement and post-intervention symptoms (37, 39, 41, 43, 47) while four studies did not find any association (45, 56, 60, 62).

### Meta-Analysis of the Effect of Engagement on Mental Health Outcomes

#### Main Analyses

Of the 25 studies included in the meta-analysis, 20 studies (20, 33–35, 37, 40, 41, 43, 47, 48, 51, 54–57, 59, 61, 63–65) employed correlational designs to investigate the association between engagement and mental health outcomes. The other five studies (36, 38, 50, 52, 66) used between-group mixed model comparisons to identify any differences in post-intervention outcomes between users who exhibited higher vs. lower levels of engagement. To maximize comparability across studies, the number of modules (also referred to as activities or sessions) completed was used as our primary measure of engagement, owing to its common use among the included studies.

**Figure 2:**
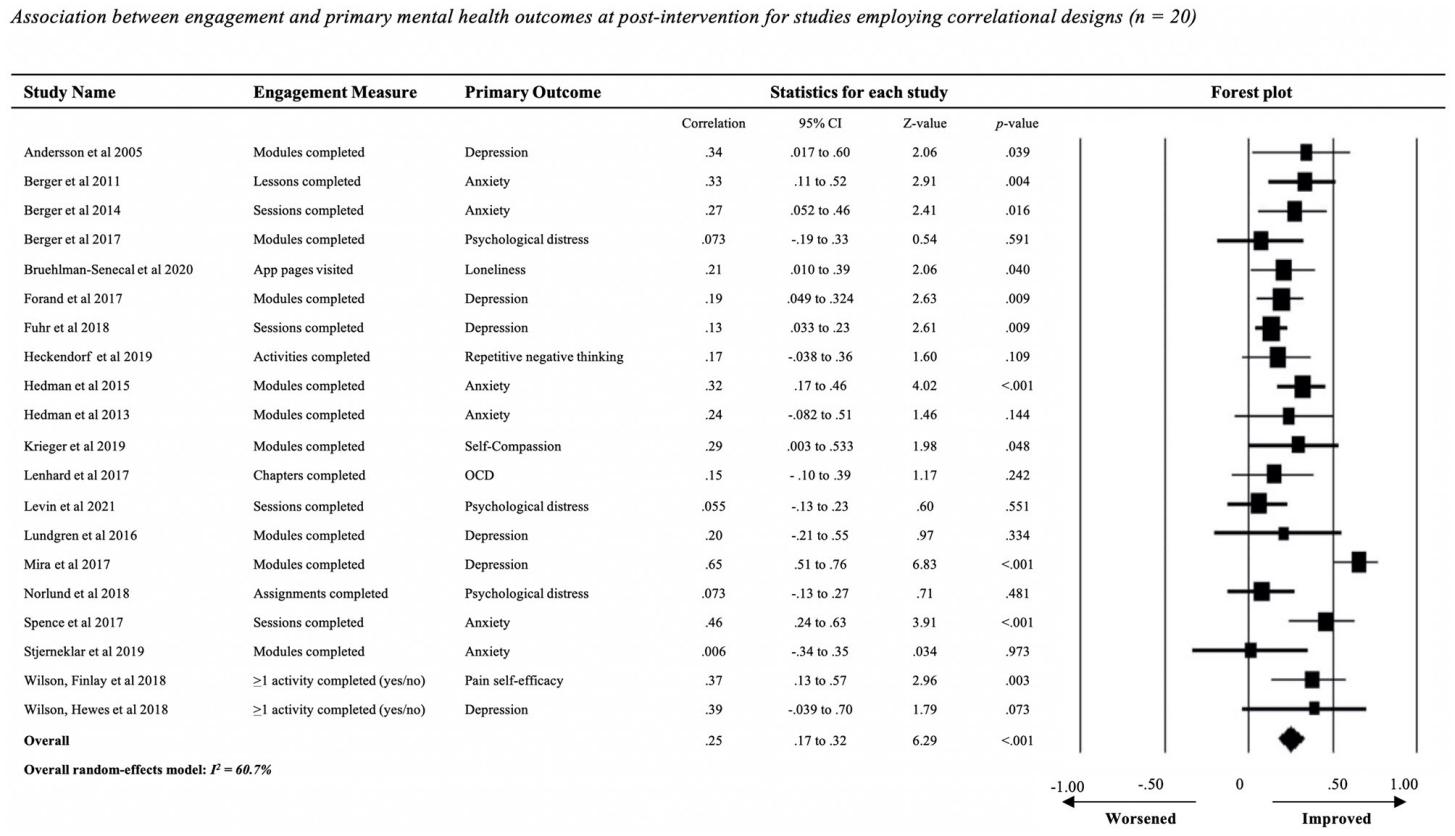
Association between engagement and primary mental health outcomes at post-intervention for studies employing correlational designs (*n* = 20).

Meta-analysis of the mean pooled correlation between number of modules completed and change in any mental health outcome (20 studies, *N* = 1,808 participants; Figure 2) showed a small, significant positive association [*r* = 0.25, 95% CI (0.17, 0.32), *Z* = 6.29, *p* < 0.001]. Leave-one-out analysis revealed that no single study rendered the random-effects model non-significant. Removal of Mira et al. (65) had the largest effect influence on the model, reducing the overall *r* from 0.25 to 0.21. There was a moderate level of heterogeneity in the distribution of individual study effect sizes (*I*^2^ = 60.7%). Examination of the funnel plot (Supplementary Figure 5A, p. 6) revealed that there was no publication bias for this analysis, as indicated by the one-tailed *p*-value (*p* = 0.11).

**Figure 3:**
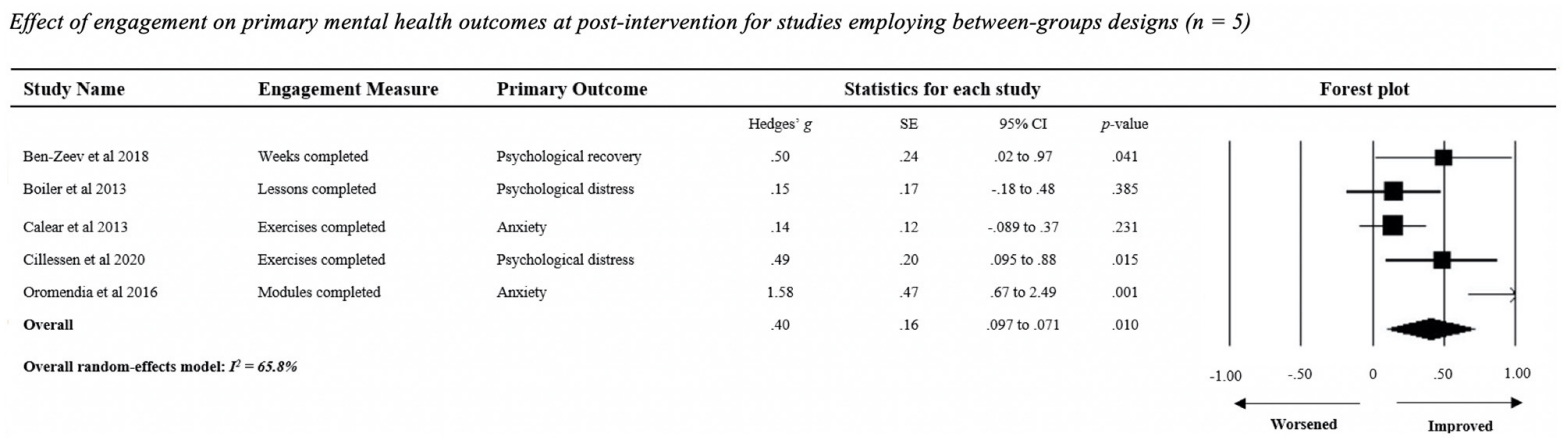
Effect of engagement on primary mental health outcomes at post-intervention for studies employing between-groups designs (*n* = 5).

Meta-analysis of the effect of engagement on any mental health outcome among studies that used between-group comparisons (*n* = 5 studies; Figure 3) showed that users reporting higher levels of content access had significant, moderate improvements in post-intervention mental health outcomes relative to users with lower levels of engagement [Hedges' *g* = 0.40, *SE* =0.16, 95% CI (0.097, 0.705), *Z* = 2.59, *p* = 0.010]. Leave-one-out analysis revealed that no single study rendered the random-effects model non-significant. Omission of Oromendia et al. (66) had the largest effect reduction, increasing the Hedges' *g* value from 0.40 to 0.25. There was a moderate level of heterogeneity in the distribution of individual study effect sizes (*I*^2^ = 65.8%).

#### Subgroup Analyses

Figures for all subgroup analyses can be found in the Supplementary Material (p. 3–5).

##### Unguided and Guided Interventions

Data for nine of the 17 studies evaluating unguided interventions and for six of the 14 studies evaluating guided interventions were examined in two separate meta-analyses. To avoid ambiguity, studies that administered interventions in both guided and unguided formats (four studies) were excluded from the analyses. The overall mean pooled correlation for the meta-analysis of nine studies evaluating unguided interventions (*N* = 674; Supplementary Figure 4A) showed a small, significant positive association between engagement and post-intervention mental health outcomes (*r* = 0.23, 95% CI [0.16, 0.31], *Z* = 6.07, *p* < 0.001). Leave-one-out analysis revealed that no single study rendered the random-effects model non-significant. Removal of Forand et al. (40) had the largest effect influence on the model, increasing the overall *r* from 0.23 to 0.25. There was minimal heterogeneity in the distribution of individual study effect sizes (*I*^2^ = 0.0%).

The overall mean pooled correlation for the meta-analysis of six studies evaluating guided interventions (*N* = 423; Supplementary Figure 4B) showed a significant, moderate positive association between engagement and post-intervention mental health outcomes (*r* = 0.30, 95% CI [0.20, 0.38], *Z* = 6.13, *p* < 0.001). Leave-one-out analysis revealed that no single study rendered the random-effects model non-significant. Removal of Spence et al. (61) had the largest effect influence on the model, reducing the overall *r* from 0.30 to 0.26. There was no heterogeneity in the distribution of individual study effect sizes (*I*^2^ = 0.0%).

##### Mental Health Diagnostic Status

We analyzed studies that used self-report symptom screening (11 studies) separately to those studies which used diagnostic instruments (six studies). The overall mean pooled correlation for the meta-analysis of self-report symptom measures (*N* = 1,056; Supplementary Figure 4C) showed a significant positive association between engagement and post-intervention mental health outcomes (*r* = 0.24, 95% CI [0.17, 0.32], *Z* = 6.29, *p* < 0.001). Leave-one-out analysis revealed that no single study rendered the random-effects model non-significant. Removal of Mira et al. (65) had the largest effect influence on the model, reducing the overall *r* from 0.27 to 0.17. There was a moderate level of heterogeneity in the distribution of individual study effect sizes (*I*^2^ = 70.2%). Examination of the funnel plot (Supplementary Figure 5B, p. 6) revealed that there was no publication bias for this analysis, as indicated by the one-tailed *p*-value (*p* = 0.08).

The overall mean pooled correlation for the meta-analysis of 6 studies with participants who fulfilled criteria for a psychiatric diagnosis (*N* = 530; Supplementary Figure 4D) showed a significant positive association between engagement and post-intervention mental health outcomes (*r* = 0.28, 95% CI [0.19, 0.36], *Z* = 6.03, *p* < 0.001). Leave-one-out analysis revealed that no single study rendered the random-effects model non-significant. Removal of Spence et al. (61) had the largest effect influence on the model, reducing the overall *r* from 0.28 to 0.26. Heterogeneity in the distribution of individual study effect sizes was minimal (*I*^2^ = 11.6%).

##### Specific Mental Health Outcomes

Data for five of the 10 studies which investigated the relationship between engagement and anxiety-related symptoms, and for six of the 12 studies involving participants who met diagnostic criteria for a mental health condition were combined and examined in two separate meta-analyses.

The overall mean pooled correlation for the meta-analysis of 5 studies with anxiety-related symptoms as the primary outcome (*N* = 411; Supplementary Figure 4E) showed a significant positive association between engagement and post-intervention mental health outcomes (*r* = 0.33, 95% CI [0.24, 0.41], *Z* = 6.76, *p* < 0.001). Leave-one-out analysis revealed that no single study rendered the random-effects model non-significant. Removal of Spence et al. (61) had the largest effect on the model, reducing the overall *r* from 0.33 to 0.20. There was no heterogeneity in the distribution of individual study effect sizes (*I*^2^ = 0.0%).

The overall mean pooled correlation for the meta-analysis of 6 studies with depressive symptoms as the primary outcome (*N* = 735; Supplementary Figure 4F) showed a significant positive association between engagement and post-intervention mental health outcomes (*r* = 0.33, 95% CI [0.13, 0.50], *Z* = 3.12, *p* = 0.002). Leave-one-out analysis revealed that no single study rendered the random-effects model non-significant. Removal of Mira et al. (65) had the largest effect on the model, reducing the overall *r* from 0.33 to 0.17. There was a high level of heterogeneity in the distribution of individual study effect sizes (*I*^2^ = 82.2%).

## Discussion

To our knowledge, this is the first systematic review and meta-analysis to quantitatively examine whether the level of user engagement with a digital intervention was associated with change in mental health outcomes after the intervention period. Although it is widely accepted that the extent of engagement with digital interventions will be positively associated with improvements in mental health, robust empirical evidence to support or validate this hypothesis is scant. While the narrative synthesis showed mixed support for a positive engagement—outcome relationship, the meta-analyses (main and subgroup) consistently supported our main a priori hypothesis. That is, the results unequivocally support that greater engagement with digital interventions is modestly but significantly associated with improvements in mental health (effect size range: *r* = 0.23 to Hedges' *g* = −0.40) regardless of the level of guidance provided, mental health symptom severity of users, or type of mental health condition(s) targeted by the intervention.

Our findings validate the qualitative findings reported in the systematic review by Donkin and colleagues (18), who reported that improvements in mental health-related outcomes appeared to be associated with the number of modules accessed, but not with other engagement indicators (e.g., time spent, logins, and online interactions). In our study, it was not possible to quantitatively explore this relationship using these latter engagement indicators as too few of the included studies reported on such data. Future studies should consider reporting associations between multiple engagement measures and mental health outcomes to continue to build the evidence base for the impact of engagement on treatment outcomes, and to reach an understanding of what level or threshold of engagement is needed to achieve therapeutic benefits.

The study findings have several important implications for clinical practice and research. Firstly, they support the view that users' level of engagement with intervention content is likely a key mechanism for predicting the amount of treatment benefit obtained (18), justifying the development of strategies aimed at increasing engagement with digital interventions. There is already some promising research being done in this space, with several studies finding that external strategies such as automated reminders (13, 67), therapist-led coaching (68, 69), and moderated peer-support groups (70) can be effective toward promoting engagement with digital interventions for mental health. Though the literature on the use of strategies is still emerging, it is worthwhile for healthcare, educational, or community-based organizations who may eventually recommend or deliver digital health interventions to consider incorporating such strategies as part of their implementation models. To ensure that digital interventions are being built in ways that users are motivated to engage with them, researchers should consider involving those with lived experience in the design and development process so that these programs are appropriately solving problems that users care about, building dynamic rather than static programs, ensuring well-integrated and meaningful gamification, and allowing personalisation or tailoring of these programs to the user (12, 71).

Secondly, our findings suggest that module completion may be one of the more acceptable measures of engagement to evaluate. Given that research on the impact of engagement on mental health outcomes has been hindered by the lack of consensus over a suitable engagement measure (72, 73), we recommend that future studies consider including module completion as the primary engagement measure to facilitate future corroboration of the present findings.

Our study had several limitations. First, the included studies differed in many ways, such as by target population, DMHIs employed, and the types of mental health conditions examined. Thus, specific analyses of the effect of a specific measure of engagement on a particular mental health outcome could not be conducted. Second, there were also differences in the statistical approaches employed by studies in how they quantified the engagement-outcome relationship. As effect sizes from repeated measures and between-subject designs are not comparable (26), data provided by both types of studies had to be analyzed separately. Third, Pearson's *r* had to be estimated for some of the studies which employed correlational designs. While estimating *r* from other indices may not be ideal, it is preferred to omitting studies without this data so as to maximize objectivity and minimize selection bias (26, 29). Finally, bivariate correlations between engagement and outcomes do not account for the possibility that other variables may influence this association. For example, one of the included studies reported that sessions completed and time spent were correlated with reduced depression scores at post-intervention; however, these associations were non-significant after email support was accounted for (63). Controlling for factors linked with engagement may be necessary for verifying the robustness of its relationship with outcomes.

This systematic review and meta-analysis provides the first meta-analytic evidence that the more that users engage with digital interventions the greater the improvements in mental health symptoms. Our findings speak to the importance of ensuring that those individuals who are less motivated to engage, or experience more barriers to engagement, have access to strategies that can overcome these challenges if we are to maximize therapeutic benefits. On a methodological level, the findings underscore the importance of standardizing measures of user engagement in future trials to build our certainty in this evidence. To further advance the field, it is important for future research to explore which engagement metrics (log-ins, sessions completed, time spent, etc.) have the greatest impact on improving mental health outcomes. This information will enable the targeted development of engagement strategies that will support users to interact with interventions in ways that they are most likely to benefit from them.

## Data Availability Statement

The raw data supporting the conclusions of this article will be made available by the authors, without undue reservation.

## Author Contributions

DZQG, MT, LM, and HC designed the study. DZQG and MT planned the statistical analysis. DZQG extracted and analyzed the data, with assistance from MT and LM. DZQG, MT, LM, and JH assessed study eligibility and quality. DZQG, MT, and LM wrote the first draft of the manuscript. All authors contributed to the interpretation and subsequent edits of the manuscript.

## Funding

DZQG was supported by an Australian Government Research Training Program Scholarship and a Centre of Research Excellence in Suicide Prevention (CRESP) Top-Up Scholarship for the completion of a PhD. MT was supported by a NHMRC Early Career Fellowship. JH was supported by a Commonwealth Suicide Prevention Research Fund Post-Doctoral Fellowship. HC was supported by a NHMRC Elizabeth Blackburn Fellowship.

## Conflict of Interest

MT, LM, JH, and HC are employed by the Black Dog Institute (University of New South Wales, Sydney, NSW, Australia), a not-for-profit research institute that develops and tests digital interventions for mental health. The remaining author declares that the research was conducted in the absence of any commercial or financial relationships that could be construed as a potential conflict of interest.

## Publisher's Note

All claims expressed in this article are solely those of the authors and do not necessarily represent those of their affiliated organizations, or those of the publisher, the editors and the reviewers. Any product that may be evaluated in this article, or claim that may be made by its manufacturer, is not guaranteed or endorsed by the publisher.
