# Supplementary material for: Effect of Engagement With Digital Interventions on Mental Health Outcomes: A Systematic Review and Meta-Analysis
Source: Front Digit Health. 2021 Nov 4;3:764079. doi: 10.3389/fdgth.2021.764079 (PMC8599127; doi:10.3389/fdgth.2021.764079)
Supplement: Supplementary file 1 [file Data_Sheet_1.DOCX]

Effect of engagement with digital interventions on mental health outcomes: A systematic review and meta-analysis

**Supplementary Material**

**Search Terms**

| MEDLINE (from 1946), PsycINFO (from 1806), EmBASE (from 1947)  till December 1, 2020. All databases accessed via OvidSP. | |
| --- | --- |
| 1 | (mental* or depress* or anx* or suicid* or self?harm or addict*).ti,ab. |
| 2 | (web* or online* or internet* or mobile* or app* or smartphone* or m?health or e?health).ti,ab. |
| 3 | (adhere* or participat* or engage* or predict*).ti,ab. |
| 4 | (randomis* or randomiz*).pt |
| 5 | 1 AND 2 AND 3 AND 4 |
| 6 | (review* or protocol*).ti,ab. |
| 7 | 5 NOT 6 |
| 8 | limit 7 to (english language and humans) |

**Cochrane Risk of Bias Ratings**

| **Studies** | | **Bias arising**  **from the randomization process** | **Bias due to deviations from the intended interventions** | **Bias due to missing outcome data** | **Bias in measurement of the outcome** | **Bias in selection of the reported result** |
| --- | --- | --- | --- | --- | --- | --- |
| Andersson et al. (2005) | | + | + | + | ? | ? |
| Ben Zeev et al. (2018) | | + | + | + | + | ? |
| Berger et al. (2011) | | ? | ? | + | ? | ? |
| Berger et al. (2014) | | + | + | ? | ? | ? |
| Berger et al. (2017) | | + | ? | + | ? | ? |
| Bolier et al. (2013) | | ? | ? | ? | ? | - |
| Bruehlman-Senecal et al. (2020) | | + | + | + | + | ? |
| Calear et al. (2013) | | + | + | + | + | + |
| Casey et al. (2017) | | + | ? | + | ? | ? |
| Cillessen et al. (2020) | | + | + | + | + | + |
| El Alaoui et al. (2013) | | + | + | + | + | ? |
| Forand et al. (2017) | | + | + | + | + | + |
| Fuhr et al. (2018) | | + | + | + | ? | + |
| Heckendorf et al. (2019) | | + | ? | + | ? | ? |
| Hedman et al. (2015) | | + | ? | + | + | ? |
| Hedman et al. (2013) | | + | + | + | + | ? |
| Hensel et al. (2019) | | + | - | - | ? | + |
| Krieger et al. (2019) | | + | ? | + | ? | ? |
| Lenhard et al. (2017) | | + | + | + | + | ? |
| Levin et al. (2016) | | ? | ? | ? | ? | ? |
| Levin et al. (2021) | | ? | + | + | + | ? |
| Lundgren et al. (2016) | | + | ? | + | ? | ? |
| Luo et al. (2021) | | ? | + | + | + | ? |
| Mira et al. (2017) | | + | ? | + | ? | ? |
| Moberg et al. (2019) | | ? | + | + | ? | ? |
| Norlund et al. (2018) | | + | + | + | + | ? |
| Oromendia et al. (2016) | | + | + | + | + | ? |
| Schlosser et al. (2018) | | ? | ? | ? | + | ? |
| Spence et al. (2017) | | + | + | + | + | ? |
| Stjerneklar et al. (2019) | | + | + | + | + | + |
| Todd et al. (2014) | | + | ? | + | ? | + |
| Twomey et al. (2018) | | ? | - | - | ? | ? |
| Wilson, Finlay, et al. (2018) | | ? | ? | ? | ? | ? |
| Wilson, Hewes, et al. (2018) | | ? | ? | ? | ? | ? |
| Zeng et al. (2020) | | + | + | + | ? | ? |
| **% with ‘Some Concerns’ or ‘High Risk’** | | 28.6% | 45.7% | 22.6% | 54.3% | 80.0% |
|  | |  |  |  |  |  |
| **Key:** | + | Low risk of bias | ? | Some Concerns | - | High risk of bias |

**Figure 4A.**

*Association between engagement and primary mental health outcomes at post-intervention for studies evaluating unguided interventions (n = 9)*


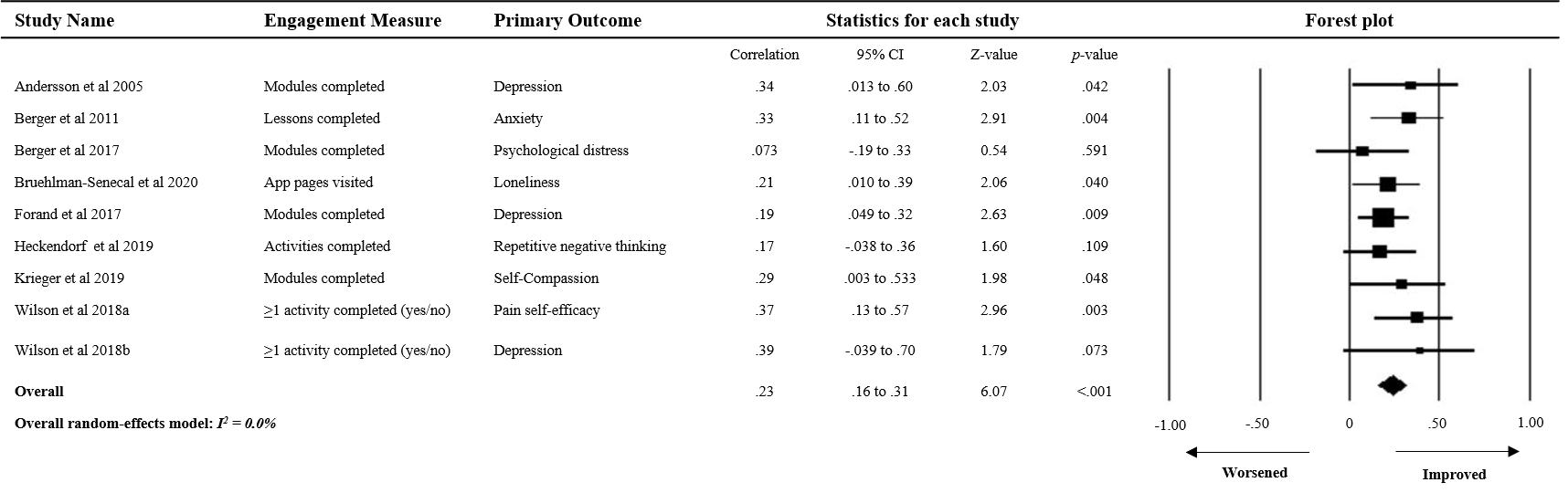


**Figure 4B.**

*Association between engagement and primary mental health outcomes at post-intervention for studies evaluating guided interventions (n = 6)*


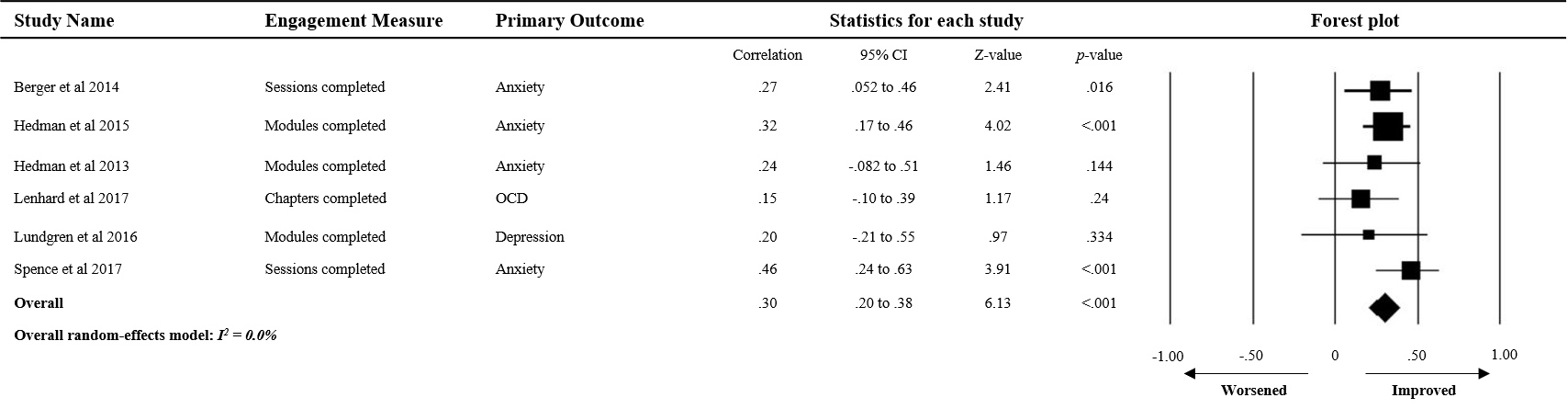


**Figure 4C.**

*Association between engagement and primary mental health outcomes at post-intervention in studies with self-reported mental health problems (n = 11)*

*
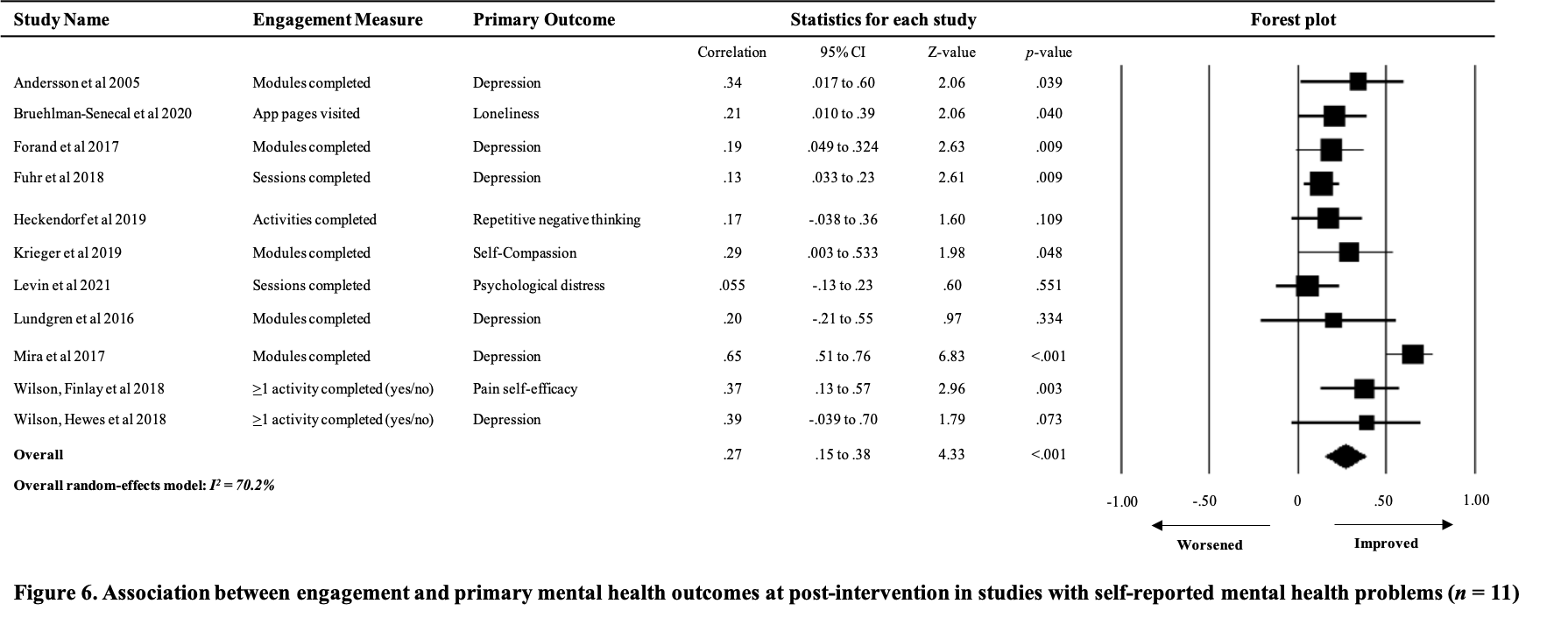
*

**Figure 4D.**

*Association between engagement and primary mental health outcomes at post-intervention in studies with participants meeting psychiatric diagnostic criteria (n = 7)*

*
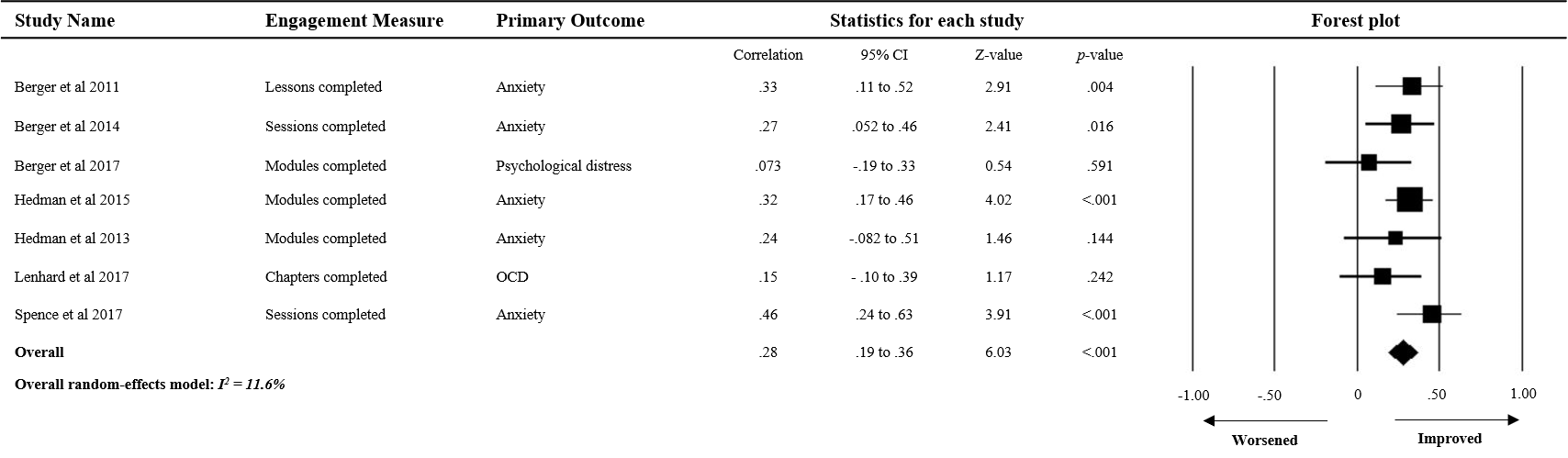
*

**Figure 4E.**

*Association between engagement and post-intervention outcomes for interventions targeting anxiety-related symptoms (n = 5)*


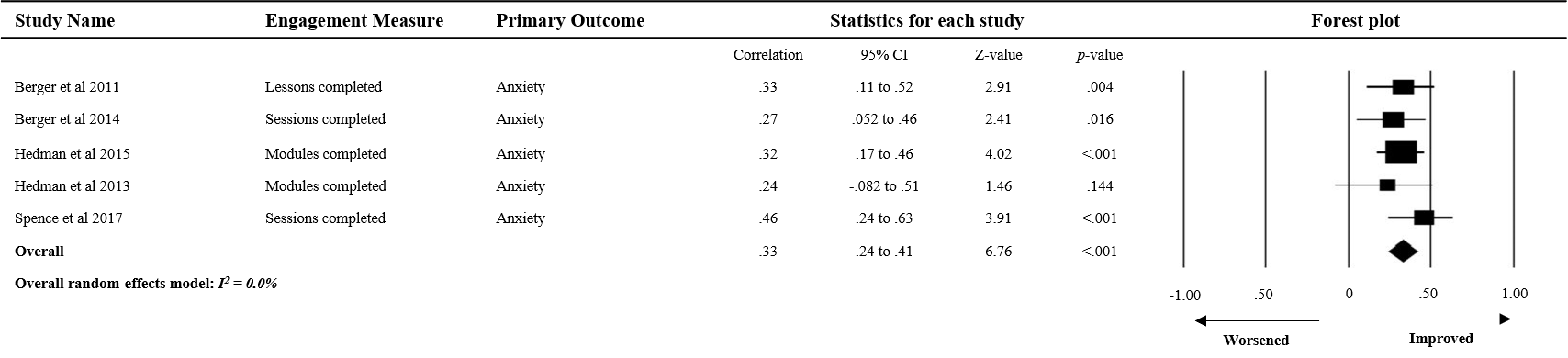


**Figure 4F.**

*Association between engagement and post-intervention outcomes for interventions targeting depression-related symptoms (n = 6)*

*
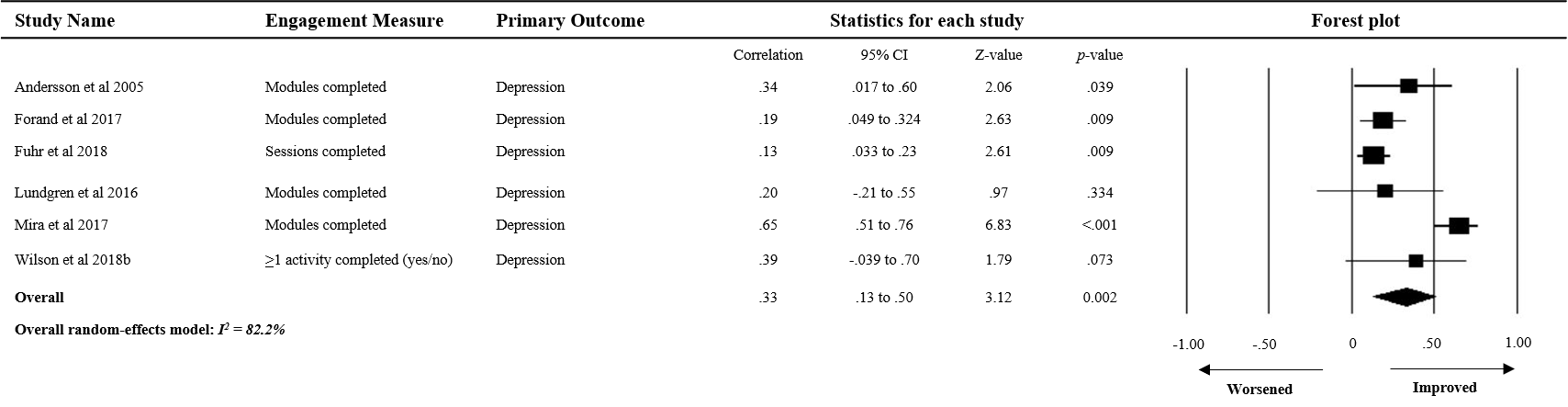
*

**Figure 5:**

*Funnel plot of studies for* (A) *all studies employing correlational designs (n = 20) and (B)* *subgroup of correlational studies with self-reported mental health problems* *(n = 11)*

**(A)**

**(B)**

Studies within the triangle have non-significant effect sizes. Studies outside the triangle are significant at the

*p* = 0.05 level. White circles denote studies included in the analysis. Black circles denote imputed studies estimated by the trim-and-fill method.

**PRISMA-P (Preferred Reporting Items for Systematic review and Meta-Analysis Protocols) 2015 checklist: recommended items to address in a systematic review protocol***

| Section and topic | Item No | Checklist item | Reported on page # of manuscript |
| --- | --- | --- | --- |
| ADMINISTRATIVE INFORMATION | | |  |
| Title: |  | **The association between engagement in digital health interventions and mental health outcomes:**  **A systematic review and meta-analysis** | 1 |
| Identification | 1a | Identify the report as a protocol of a systematic review | N/A |
| Update | 1b | If the protocol is for an update of a previous systematic review, identify as such | N/A |
| Registration | 2 | If registered, provide the name of the registry (such as PROSPERO) and registration number | PROSPERO; CRD42020184706 |
| Authors: |  |  |  |
| Contact | 3a | Provide name, institutional affiliation, e-mail address of all protocol authors; provide physical mailing address of corresponding author | 1 |
| Contributions | 3b | Describe contributions of protocol authors and identify the guarantor of the review | 10 |
| Amendments | 4 | If the protocol represents an amendment of a previously completed or published protocol, identify as such and list changes; otherwise, state plan for documenting important protocol amendments | N/A |
| Support: |  |  |  |
| Sources | 5a | Indicate sources of financial or other support for the review | 11 |
| Sponsor | 5b | Provide name for the review funder and/or sponsor | N/A |
| Role of sponsor or funder | 5c | Describe roles of funder(s), sponsor(s), and/or institution(s), if any, in developing the protocol | N/A |
| INTRODUCTION | | |  |
| Rationale | 6 | Describe the rationale for the review in the context of what is already known | 1-3 |
| Objectives | 7 | Provide an explicit statement of the question(s) the review will address with reference to participants, interventions, comparators, and outcomes (PICO) | 3 |
| METHODS | | |  |
| Eligibility criteria | 8 | Specify the study characteristics (such as PICO, study design, setting, time frame) and report characteristics (such as years considered, language, publication status) to be used as criteria for eligibility for the review | 3-4 |
| Information sources | 9 | Describe all intended information sources (such as electronic databases, contact with study authors, trial registers or other grey literature sources) with planned dates of coverage | 3 |
| Search strategy | 10 | Present draft of search strategy to be used for at least one electronic database, including planned limits, such that it could be repeated | Supp.  Material p.1 |
| Study records: |  |  |  |
| Data management | 11a | Describe the mechanism(s) that will be used to manage records and data throughout the review | 4-5 |
| Selection process | 11b | State the process that will be used for selecting studies (such as two independent reviewers) through each phase of the review (that is, screening, eligibility and inclusion in meta-analysis) | 4-5 |
| Data collection process | 11c | Describe planned method of extracting data from reports (such as piloting forms, done independently, in duplicate), any processes for obtaining and confirming data from investigators | 4-5 |
| Data items | 12 | List and define all variables for which data will be sought (such as PICO items, funding sources), any pre-planned data assumptions and simplifications | 4-5 |
| Outcomes and prioritization | 13 | List and define all outcomes for which data will be sought, including prioritization of main and additional outcomes, with rationale | 3-4 |
| Risk of bias in individual studies | 14 | Describe anticipated methods for assessing risk of bias of individual studies, including whether this will be done at the outcome or study level, or both; state how this information will be used in data synthesis | 5 |
| Data synthesis | 15a | Describe criteria under which study data will be quantitatively synthesised | 4-5 |
|  | 15b | If data are appropriate for quantitative synthesis, describe planned summary measures, methods of handling data and methods of combining data from studies, including any planned exploration of consistency (such as I^2^, Kendall’s τ) | 4-5 |
|  | 15c | Describe any proposed additional analyses (such as sensitivity or subgroup analyses, meta-regression) | 4-5 |
|  | 15d | If quantitative synthesis is not appropriate, describe the type of summary planned | NA |
| Meta-bias(es) | 16 | Specify any planned assessment of meta-bias(es) (such as publication bias across studies, selective reporting within studies) | 5 |
| Confidence in cumulative evidence | 17 | Describe how the strength of the body of evidence will be assessed (such as GRADE) |  |

*** It is strongly recommended that this checklist be read in conjunction with the PRISMA-P Explanation and Elaboration (cite when available) for important clarification on the items. Amendments to a review protocol should be tracked and dated. The copyright for PRISMA-P (including checklist) is held by the PRISMA-P Group and is distributed under a Creative Commons Attribution Licence 4.0.**

*From: Shamseer L, Moher D, Clarke M, Ghersi D, Liberati A, Petticrew M, Shekelle P, Stewart L, PRISMA-P Group. Preferred reporting items for systematic review and meta-analysis protocols (PRISMA-P) 2015: elaboration and explanation. BMJ. 2015 Jan 2;349(jan02 1):g764*
